# Exploring Biosurfactant Production from Halophilic Bacteria, Isolated from Burgas Salterns in Bulgaria

**DOI:** 10.3390/md24010053

**Published:** 2026-01-22

**Authors:** Kaloyan Berberov, Ivanka Boyadzhieva, Boryana Yakimova, Hristina Petkova, Ivanka Stoineva, Lilyana Nacheva, Lyudmila Kabaivanova

**Affiliations:** 1 Laboratory of Extremophilic Microorganisms, Department of General Microbiology, Institute of Microbiology–Bulgarian Academy of Sciences, 1113 Sofia, Bulgaria; kberberov@microbio.bas.bg (K.B.); petrovaim@abv.bg (I.B.); 2 Competence Center “Clean Technologies for Sustainable Environment–Water, Waste, Energy for Circular Economy”, Sofia University, 1164 Sofia, Bulgaria; 3 Laboratory of Chemistry and Biophysics of Proteins and Enzymes, Institute of Organic Chemistry with Centre of Phytochemistry, Bulgarian Academy of Sciences, 1113 Sofia, Bulgaria; boryana.yakimova@orgchm.bas.bg (B.Y.); ivanka.stoineva@orgchm.bas.bg (I.S.); 4 Institute of Neurobiology, Bulgarian Academy of Sciences, 1113 Sofia, Bulgaria; 5 Institute of Physical Chemistry ‘Rostislaw Kaischew’, Bulgarian Academy of Sciences, 1113 Sofia, Bulgaria; hpetkova@ipc.bas.bg; 6 Laboratory of Bioremediation and Biofuels, Department of Biotechnology, Institute of Microbiology–Bulgarian Academy of Sciences, 1113 Sofia, Bulgaria; lin1@abv.bg

**Keywords:** biosurfactant, halophiles, halophilic bacteria, *Halomonas*, hexadecane, lipopeptide, salterns

## Abstract

Biosurfactants produced by halophilic bacteria are gaining attention as eco-friendly and biocompatible alternatives to synthetic surfactants due to their high surface activity, stability under extreme conditions, and intrinsic antimicrobial properties. These amphiphilic biomolecules hold great promise for bioremediation, biomedical, and pharmaceutical applications. In this study, moderately halophilic bacteria capable of biosurfactant production were isolated from saline mud collected at the Burgas solar salterns (Bulgaria). The halophilic microbiota was enriched in Bushnell–Haas (BH) medium containing 10% NaCl amended with different carbon sources. Primary screening in BH liquid medium evaluated the isolates’ ability to degrade n-hexadecane while at the same time producing biosurfactants. Thirty halophilic bacterial strains were isolated on BH agar plates supplemented with 2% n-hexadecane, 2% olive oil, or 2% glycerol. Four isolates—BS7OL, BS8OL, BS9GL, and BS10HD—with strong emulsifying activity (E_24_ = 56%) and reduced surface tension in the range of 27.3–45 mN/m were derived after 7 days of batch fermentation. Strain BS10HD was chosen as the most potent biosurfactant producer. Its phylogenetic affiliation was determined by 16S rRNA gene sequence analysis; according to the nucleotide sequence, it was assigned to *Halomonas ventosae*. The extract material was analysed by thin-layer chromatography (TLC) and Fourier transform infrared spectroscopy (FTIR). Upon spraying the TLC plate with ninhydrin reagent, the appearance of a pink spot indicated the presence of amine functional groups. FTIR analysis showed characteristic peaks for both lipid and peptide functional groups. Based on the observed physicochemical properties and analytical data, it can be suggested that the biosurfactant produced by *Halomonas ventosae* BS10HD is a lipopeptide compound.

## 1. Introduction

Biosurfactants (BSs) are amphiphilic molecules predominantly produced by microorganisms. Their structure consists of hydrophilic and hydrophobic moieties. This structural feature enables biosurfactants to reduce the surface and interfacial tension between individual molecules at the surface and between two interfaces, respectively. Due to their amphiphilic properties, BSs are extensively used as emulsifiers, detergents, dispersants, and wetting and foaming agents [1] and they find applications in the pharmaceutical, medicine, food, and agricultural industries [2].

Microbial biosurfactants include a wide range of chemical structures. The low-molecular-weight BSs include glycolipids (rhamno-, sophoro- and trehalolipids), phospholipids, and lipopeptides; polymeric compounds such as polysaccharides, lipopolysaccharides, lipoproteins, and proteins represent high-molecular-weight BSs [3]. Glycolipids and lipopeptides are preferentially studied because of their low molecular mass and the versatility of their functions (antimicrobial, anticancer, foaming, and emulsifying activities) [4]. In general, biosurfactants are used as eco-friendly alternatives of the synthetic surfactants due to their biodegradability and biocompatibility. Compared to the petrochemically and oleochemically synthesized surfactants, BSs are non-toxic to the environment and do not pose environmental risks [5]. Moreover, bacterially produced BSs have been used for bioremediation of oil-polluted environments with extreme salinity, acidity, or temperatures [6].

Extremophilic bacteria are unique due to their capability of surviving in such extreme environments, revealing the resilience of life on Earth [7]. They can survive low or high temperatures, high pressures, high salinity, low oxygen levels, high radiation, and high acidity, or alkalinity, which are conditions that would kill other forms of life. Extremophiles must develop adaptive mechanisms and strategies for coping with these harsh environmental challenges. In order to adapt to these environments, they undergo biochemical and physiological adaptations, leading to the production of extremolites, extremozymes, polymers, biosurfactants, etc. [8]. Recent studies unveiled that these secondary metabolites exhibit remarkable bioactivity under extreme conditions, making them suitable for industrial use, where harsh physicochemical conditions are typically encountered [9]. Thermophilic, psychrophilic, halophilic, and acidophilic bacteria are now largely investigated for their capability for biosurfactant production [6]. A few studies have reported on biosurfactants originating from halophilic bacteria—dwelling in various saline environments (sea water, salterns, salt lakes, salt mines, etc.) characterized by elevated concentration of salts.

Most of the halophilic BS producers were found to fall in the *Halomonas* [10,11,12,13,14,15], *Bacillus* [16], *Marinobacter* [17], *Rhodococcus* [18], *Pseudomonas* [19,20,21], and *Psychrobacter* genera [22]. Glycolipids, lipopeptides, and polymeric substances had been isolated from *Halomonas* sp. BS4 and further characterized to process antibacterial and antitumour activity [14]. *Halomonas* sp. MB-30 isolated from a marine sponge was also found to synthesize a glycolipid biosurfactant [12]. A biosurfactant-producing halophile was isolated from the brackish water of Lake Chilika and identified as *Bacillus tequilensis* CH. The structure of the biosurfactant was partially characterized as a lipopeptide, showing the inhibition of pathogenic biofilm on hydrophilic and hydrophobic surfaces [23]. Biosurfactants production by SM-23 strain of *Virgibacillus*, identified as *Virgibacillus massiliensis*, was demonstrated by Essghaier et al. (2024) [24], proving novel lipopeptides production. More intriguing is the ability of halophiles to couple hydrocarbons degrading with biosurfactant production. Utilization of these substrates appears as to be a promising alternative for valorising these wastes, thereby reducing production costs and promoting environmental sustainability. Crude oil hydrocarbons are strong environmental pollutants that cause health problems by contaminating water, soil, and air [25]. Halotolerant *Halomonas* sp. C2SS100 was able to degrade crude oil combined with biosurfactant production [15]. The ability of *Halomonas* sp. KHS3 to degrade aromatic compounds was investigated by Monzon et al. (2018) [11]. The strain was capable of growing on fluorene and naphtalene as the only carbon sources while synthesizing rhamnolipids. The bioconversion of glycerol and n-hexadecane to glycolipids was reported for *Halomonas elongata* BK-AG18 [10] and *Halomonas* sp. Ant-3b [13], respectively, indicating that various hydrocarbon substances could be used as substrates for biosurfactant production.

In this study, we aimed to isolate halophilic bacteria capable of biosurfactant production coupled with hydrocarbon degradation. Out of 30 strains, *Halomonas ventosae* BS10HD showed good emulsifying activity and low surface tension in its cell-free supernatant. The strain was able to grow on different carbon sources. The biosurfactant production was optimized and the surface-active agent was further characterized.

## 2. Results

### 2.1. Isolation of Halophilic Bacteria Capable of Growing on Different Carbon Sources

Some of the physicochemical parameters (pH, temperature, and salinity) of the saline mud sample designated as BST5 were measured in the field. The pH of the sample was 7.5. The salinity was measured to be 24%; its temperature was 36.8 °C. Colonies developed from only 10^−2^ to 10^−5^ dilutions, made from the cultures that underwent two rounds of enrichment in modified BH medium. A total of 30 colonies were chosen based on their distinct macromorphological characteristics and these were re-streaked several times on the same culture medium in order to obtain pure cultures. Ten strains were isolated from each type of enrichment culture with 2% n-hexadecane (strains BS1HD–BS10HD), 2% olive oil (strains BS1OL–BS10OL), or 2% glycerol (strains BS1GL–BS10GL). The isolates are summarized in Table 1. These strains were subjected to biosurfactant screening.

### 2.2. Screening for Biosurfactant Production from the Isolates

The possible biosurfactant production by the isolates was assayed using CFSs obtained from liquid cultures in BH medium as shown in Table 1. Initially, the oil displacement test was used to investigate the presence of amphiphilic molecules in the obtained CFSs. The oil-displacement zones made in the olive oil layer were in the range of 2–30 mm. Most of the CFSs (57%) demonstrated zones between 10 mm and 24 mm, indicating biosurfactant production. One third of the investigated CFSs manifested very weak displacement zones in the range of 2–5 mm. A parafilm M assay was also conducted in order to screen the CFSs for presence of compounds able to reduce the contact angle between the CFS drop and the hydrophobic surface. Only nine CFSs were found to demonstrate flat-shaped drops. The haemolytic activity of the CFSs was investigated with blood agar plates which could give clues for synthesis of lipopeptides or glycolipids [4]. The observed haemolysis zones varied between 5–10 mm. Most of the obtained CFSs (57% of them) produced 5–7 mm halo zones of beta-haemolysis. Ten of these did not demonstrate haemolytic activity. Finally, the emulsifying activity of the produced CFSs was evaluated. Most of the CFSs gave low E_24_ indices from 43% to 50%, comparable to that of the uninoculated BH medium (35%). Only four CFSs from strains BS7OL, BS8OL, BS9GL, and BS10HD showed a promising E_24_ index of 56.25%.

**Figure 1:**
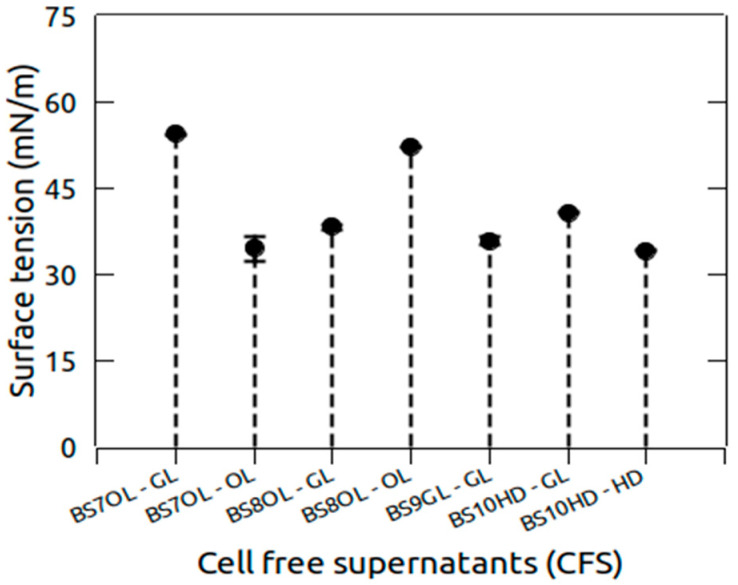
Surface tension decreased in CFSs from the prioritized strains. GL—glycerol; OL—olive oil; HD—n-hexadecane. The CFSs were obtained from liquid cultures in which the strains were grown on glycerol and the respective carbon source on which they were enriched in the previous assay. Error bars indicate the standard deviation.

These four strains were isolated after enrichment with different carbon sources (glycerol, olive oil, or n-hexadecane) and were subsequently screened under standardized conditions in BH medium supplemented with glycerol. After a batch fermentation process the surface tension of the CFSs obtained from each type of liquid culture was estimated. The testing confirmed the possible biosurfactant production from these strains as could be seen from the surface tension decrease in Figure 1. The highest decline in the surface tension (34.1 mN/m) was measured for strain BS10HD, grown on n-hexadecane. Only combinations of isolates and substrates with good growth, surface activity and reduction in surface tension are shown. 

### 2.3. Isolates Identification and Phylogenetic Analysis

**Figure 2:**
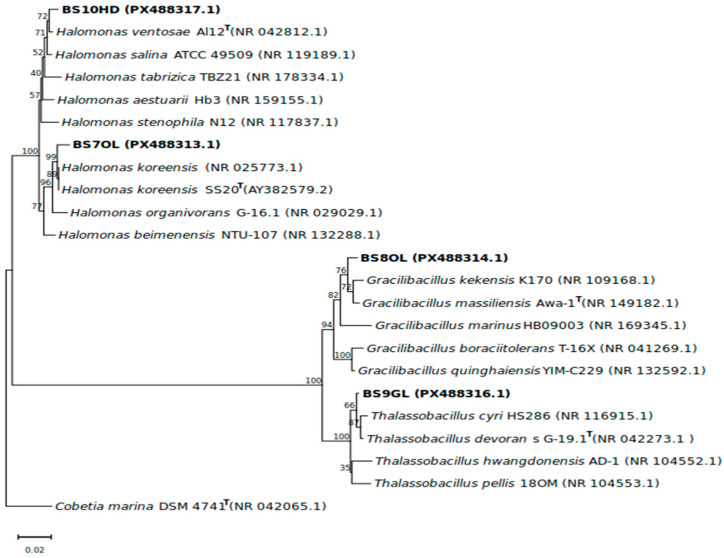
Reconstructed neighbour-joining phylogenetic tree showing the phylogenetic relationships of the isolates with their closest relatives. The values shown above the branches present the bootstrap values after 1000 replications. The legend depicts 0.02 substitutions per nucleotide site as a unit of distance. *Cobetia marina* DSM 4741^T^ was used as an outgroup to root the tree.

Based on the initial biosurfactant screening, four strains, namely BS7OL, BS8OL, BS9GL, and BS10HD, were chosen as the most potent for biosurfactant production. They were identified based on the sequence of the 16S rRNA gene. As can be seen in Table 2, two of them were affiliated to the *Halomonas* genus within the *Pseudomonadota* phylum. BS7OL and BS10HD were identified as *H. koreensis* and *H. ventosae*, respectively. The other two strains were assigned to the *Bacillota* phylum − BS8OL was identified as *Gracilibacillus massilensis* and BS9GL was identified as *Thalassobacillus devorans.* All of the obtained 16S rRNA sequences showed more than 98.65% sequence similarity [26] compared to the type strains of the respective species and formed stable branches with them that were distinct from those of the other species in the same genus (Figure 2).

### 2.4. Optimal Fermentation Conditions for Biosurfactant Production by H. ventosae BS10HD

**Figure 3:**
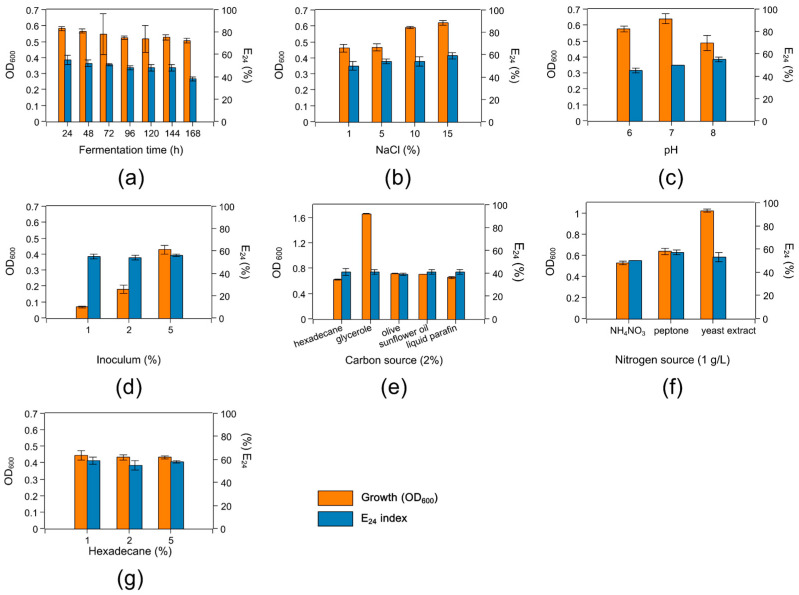
Effect of some fermentation conditions on the growth of *H. ventosae* BS10HD: (**a**) fermentation time; (**b**) NaCl concentration; (**c**) pH of BH medium; (**d**) inoculum concentration; (**e**) carbon source type; (**f**) nitrogen source type, and (**g**) n-hexadecane concentration. The effects of each parameter were measured by OD_600_ and the emulsifying activity expressed by E_24_ of the CFS obtained from liquid cultures of the strain. Error bars indicate the standard deviation.

Because of the lowest surface tension of the CFS of *H. ventosae* BS10HD, we chose this strain for the optimization of the biosurfactant production. The fermentation time, NaCl concentration, pH of BH medium, inoculum volume, carbon and nitrogen source type, and n-hexadecane concentration were varied (Figure 3). The growth of the strain measured by OD_600_ and the emulsifying activity (as E_24_ index) were used as indicators for the effect of the various conditions on the biosurfactant production. Fermentation time did not significantly influence the growth or the E_24_ index. After 24 h, the growth and E_24_ index were maximal; after that, both slightly declined until 120 h. Maximal growth and E_24_ index were also observed at 15% NaCl, 5% inoculum, and 1% n-hexadecane. The best nitrogen source proved to be peptone based on the highest emulsifying activity of E_24_ = 57%. The optimal pH of the BH medium was pH 8. The type of carbon source did not have effect on the emulsifying activity, but glycerol led to maximal cell growth. However, for all carbon sources, E_24_ was low, in the range of 39–41%.

**Figure 4:**
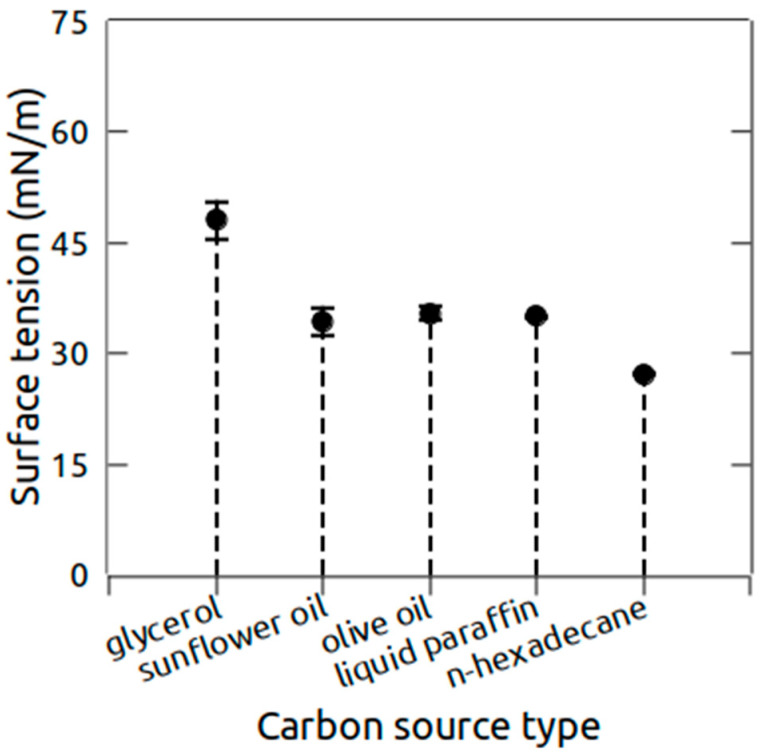
Influence of the carbon source on the surface tension decrease in the CFS of *H. ventosae* BS10HD. Error bars indicate the standard deviation.

The influence of the carbon source type on the biosurfactant production was also studied. *H. ventosae* BS10HD was grown on five different carbon sources (olive oil, glycerol, sunflower oil, liquid paraffin, and n-hexadecane) and the surface tension of the CFS from each culture was measured (Figure 4). The value (48.5 ± 2.5 mN/m) was observed for glycerol. When olive oil, sunflower oil, and liquid paraffin were used, the surface tension was relatively equal, in the range of 34–35 mN/m. The maximal decrease in the surface tension (27.25 ± 0.2) was registered with n-hexadecane as a carbon source.

### 2.5. Biosurfactant Production Kinetics of H. ventosae BS10HD

**Figure 5:**
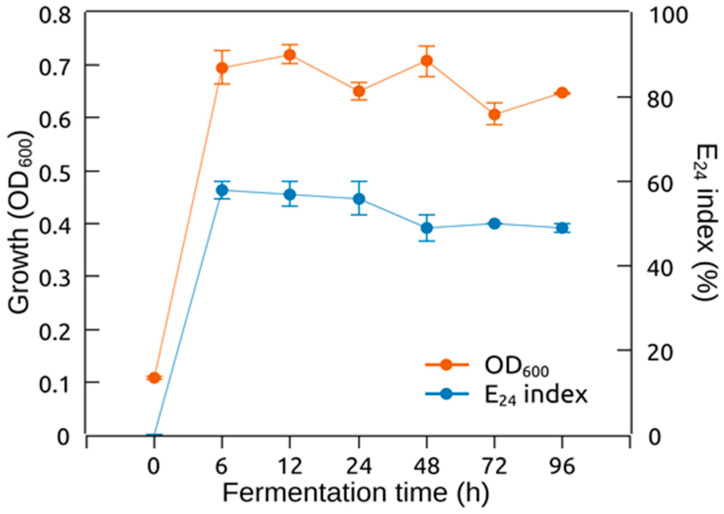
Growth and biosurfactant production profile of *H. ventosae* BS10HD. Error bars indicate the standard deviation.

The growth of *H. ventosae* BS10HD and the emulsifying activity of its CFS were monitored over a period of 96 h. The culture reached maximal optical density 6 h after inoculation. From the 12th to the 96th hours during the stationary phase of growth, the OD_600_ was stable. Maximum E_24_ index (58%) was observed at the 6th hour of cultivation. After this period, the emulsifying index declined until the 96th hour, where it was 49%. According to the data, biosurfactant production was associated with the growth of *H. ventosae* BS10HD. The production was mainly conducted in the exponential phase of growth, as can be seen in Figure 5.

### 2.6. Biosurfactant Extraction

Cell-free supernatants were extracted using various solvents, including ethyl acetate, methylene chloride, and chloroform, to identify the most effective solvent for biosurfactant recovery. Methylene chloride was selected as the most suitable solvent based on the apparent recovery of crude biosurfactant, as assessed by a visual inspection of the residue obtained after solvent evaporation. At this stage of the study, the biosurfactant yield was not quantitatively determined.

### 2.7. Thin-Layer Chromatography (TLC)

**Figure 6:**
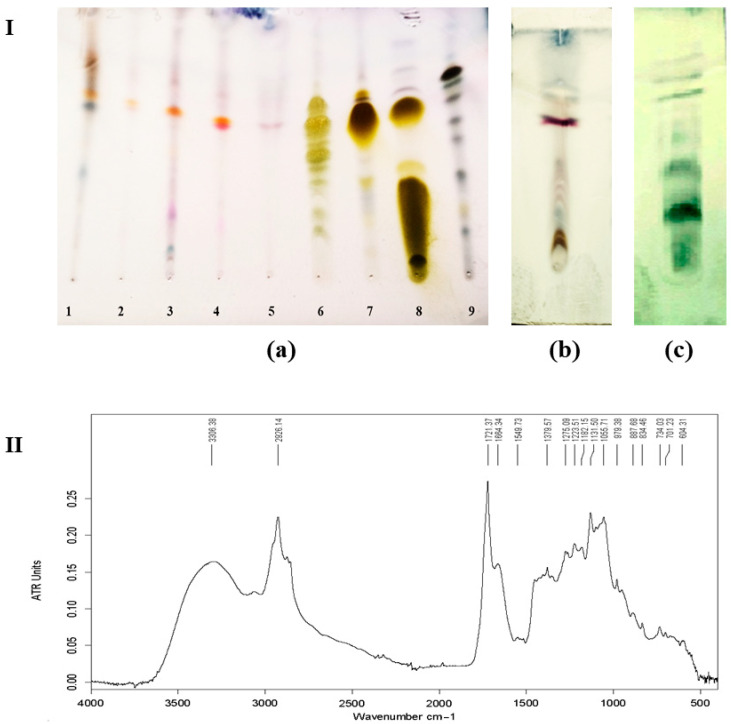
(**I**) Thin-layer chromatography (TLC) on silica gel (0.25 mm, F254, Merck) eluted with CHCl_3_/MeOH/H_2_O (65:25:2, by vol.): (**a**) 1—BS10HD grown on n-hexadecane; 2—BS10HD grown on olive oil; 3—BS10HD grown on sunflower oil; 4—BS10HD grown on glycerole; 5—BS10HD grown on liquid paraffin; 6—trehalolipid; 7—mono-rhamnolipid; 8—mono- + di-rhamnolipid; 9—sophorolipid. The spots were visualized with 2% anthrone: (**b**) BS10HD grown on n-hexadecane, where spots were visualized with 2% ninhydrine; (**c**) BS10HD grown on n-hexadecane, where spots were visualized with phosphomolybdic acid. (**II**) FTIR spectrum of the biosurfactant produced by *H. ventosae* BS10HD.

Biosurfactant analysis by TLC using specific staining reagents revealed several spots with different retention factor (Rf) values. In this study, upon visualization with 2% anthrone reagent, the typical yellow–green spots observed for the glycolipid-positive controls (rhamnolipids, trehalose lipids, and sophorolipids) was absent for crude samples 1–5 (see Figure 6 (Ia)). This indicates that the five crude surface-active compounds did not possess a carbohydrate moiety.

The presence of dark blue spots on the TLC results shown in Figure 6I(c), resulting from spraying with phosphomolybdic acid, was an indicator of the presence of fatty acids/lipids in the structure of the tested compound. The detection of both peptide and lipid moieties suggests that *H. ventosae* BS10HD produced lipopeptide-type biosurfactants when cultivated in a medium containing *n*-hexadecane as the carbon source.

### 2.8. Fourier-Transform Infrared Spectroscopy Analysis

The chemical structure of the biosurfactant compounds produced by *H. ventosae* BS10HD was determined by the identification of functional groups. FTIR spectra exhibited strong absorption over 3250 cm^−1^ to 3500 cm^−1^, with maxima at 3306 cm^−1^, which was due to –N–H, C–H, and –OH stretching, indicating the presence of carbon-containing compound with and amino group. Absorption at this range also implies the presence of intermolecular hydrogen bonds [27].

The bands at 2926.14 cm^−1^ and 1379.57 cm^−1^ implied the presence of aliphatic chains (C–H stretching mode), while the peak at 1275.09 cm^−1^ showed the C–O deformation vibrations. A band at 1549.73 cm^−1^ indicated the presence of C=O bond in the biosurfactant, which might have been caused by the C=O stretching vibrations [28]. The band at 1223.51 cm^−1^ and 1721.37 cm^−1^ was due to the lactone carbonyl absorption, which indicated that the sample was a kind of lipopeptide compound [29].

The results of the TLC analysis using ninhydrin as a colorimetric reagent for the detection of amino acids, peptides, and amine-containing compounds indicated the presence of such functional groups in the analysed samples. The absence of a carbohydrate moiety further suggests that the newly obtained compounds are not glycolipids. Based on the observed physicochemical properties, including a reduction in surface tension to 27.25 mN/m, together with FTIR analytical data (Figure 6II), it can be suggested that the biosurfactant produced by *H. ventosae* BS10HD is a lipopeptide compound.

## 3. Discussion

Following the interest in environmentally friendly surfactants that can resist extreme conditions, this study evidences the importance of exploring extremophilic microorganisms as their producers. The new market appeal for sustainable products and industrial processes can be met by the involvement of biosurfactants and continuation of mining their unique biotechnological potential [6]. The marine environment, rich in biological diversity, comprises over 70% of Earth’s surface. Many researchers have investigated this niche, searching for the producers of biologically active substances such as biosurfactants, which are applicable in different areas [14,30,31]. Eight distinct halophilic bacteria isolated from extreme saline soil samples from Salt Mines in Pakistan were investigated and proved biosurfactant production [32]. There are different techniques for obtaining crude extracts that are free of aqueous culture media. The extraction technique for lipopeptide biosurfactants from strains of *Bacillus* is a combination of acid precipitation and solvent extraction with methanol [33]. The most commonly reported method for the extraction of *Pseudomonas* lipopeptides is solvent extraction with ethyl acetate [34]. A mixture of chloroform/methanol (60:40, *v*/*v*) was used for the extraction of the surface-active compound with lipopeptide nature from the cell-free supernatant of *Halomonas venusta* strain [35]. In our case, it was established that the highest yield of crude biosurfactant was obtained by extraction with methylene chloride of the supernatant of strain *Haomonas ventosae* BS10HD. Which organic solvent is the most appropriate for removing the targeted product from the culture supernatant may depend on the producer strain and the precise structure of the lipopeptide biosurfactant.

Pharmacologically important biosurfactants were isolated and characterized from the halophilic *Bacillus* sp. BS3 [16]. In this study, different screening assays were performed for the identification of biosurfactant producers from a saline environment. From thirty halophilic bacterial strains, four were picked showing strong emulsifying activity (E_24_ = 56%) and reduced surface tension in the range of 27.3–45 mN/m after 7 days of batch fermentation. Similar values were reported for a biosurfactant produced by the *Halomonas* sp. MB-30 strain which reduced the surface tension to 30 mN/m in both glucose and hydrocarbon-supplemented minimal media [12]. Different alternatives increase biosurfactants yield; it is important to remember here that the nutritional factors required are different among microorganisms [36]. A variety of wastes generated from industries and processing plants could serve as potential candidates as carbon sources for biosurfactant production, which are abundantly available compared to the pure costly substrates. Maximal growth and E_24_ index were observed at 15% NaCl, 5% inoculum, and 1% n-hexadecane for the newly isolated *Halomonas ventosae*. *Halobacterium jilantaiense* strain JBS1 was reported to be the most effective strain with the best oil displacement test and emulsification activity against kerosene and crude oil [37]. Another study mentions that *Halomonas* sp. MM93 can be used to produce a low-molecular-weight polymer that lowers the surface tension to 40 mN/m [38]. The different types of biosurfactants—peptides, fatty acids, phospholipids, glycolipids, antibiotics, and polymers—all have advantages over their synthetic counterparts [22]. Among these different types, the biosurfactant produced by the newly isolated halophilic strain *H. ventosae* BS10HD was revealed as a lipopeptide compound, confirmed by TLC and FTIR analyses. This is in accordance with the results obtained from Cheffi et al. (2020) [39] about the biosurfactant isolated from the strain *Halomonas pasifica*. TLC data revealed a spot with Rf 0.72 when the plate was visualized with 2% ninhydrine. Considering the Rf value, the lack of a carbohydrate moiety, and positive ninhydrin test of the investigated crude samples was inferred to contain a peptide moiety and was therefore identified as a lipopeptide. The results, indicating the lipopeptide nature of the biosurfactant produced by *H. ventosae* BS10HD, identify the strain as one of the few representatives of the *Halomonas* genus, synthesizing low-molecular-weight amphiphilic lipopeptides with pronounced surface activity. Bacillus lipopeptides possess a broad spectrum of biological activities such as antimicrobial, antitumour, immunosuppressant, and antidiabetic [40,41]. Lipopeptide surfactants are also applied in the bioremediation process for maintaining sustainable environment [42]. Optimal growth on n-hexadecane is an important indicator of the bioremediation potential of halophilic bacteria, since n-hexadecane is a representative of the long-chain alkanes constituting a significant portion of crude oil. The ability to grow on n-alkanes is often associated with the induction of biosurfactant production, as biosurfactants increase the solubility, transportability, and bioavailability of hydrophobic substrates by solubilization, emulsification, and reduction in surface and interfacial tension [43]. Besides the bioremediation of hydrocarbons and microbial enhanced oil recovery, these valuable bioproducts have potential uses in the agriculture, cosmetics, pharmacy, detergent, food, textile, paper, and paint industries [44].

## 4. Materials and Methods

### 4.1. Reagents and Chemicals

Bacteriological agar powder and peptone were purchased from HiMedia (HiMedia Laboratories GmbH, Odenwald str. 18 A, 64,397 Modautal, Germany). Yeast extract was obtained from Carl Roth GmbH + Co. KG (Karlsruhe, Germany). Olive oil and triolein were purchased from Fisher Scientific (Hampton, NH, USA). Hexadecane was obtained from Sigma-Aldrich (Merck KGaA, Darmstadt, Germany). All other reagents were purchased from Sigma-Aldrich (Merck KGaA, Darmstadt, Germany) and Thermo Fisher Scientific Inc. (Waltham, MA, USA), unless otherwise stated.

### 4.2. Sampling

A sample of saline mud was aseptically collected from the Burgas salterns situated near Burgas town, Bulgaria (42.529845° N; 27.487366° E). Sampling took place in August 2024. The sample was collected using a sampling pole equipped with a sterile 100 mL polypropylene container at the end of the pole, transferred in sterile 50 mL Falcon tube, and transported to the laboratory at 4 °C in a cooler bag (VEVOR^®^ Car Refrigerator 20 L, Indespale Technology Limited, Dublin, Ireland) within 6 h after sampling. In the laboratory, the sample was stored at 4 °C in a refrigerator until use. The temperature, pH, and salinity of the sample were measured at the field with a portable pH meter (pH CHECK, Carl Roth GmbH Co. KG, Karlsruhe, Germany) and portable handheld refractometer HR-190G (OPTIKA^®^, Ponteranica, Italy), respectively.

### 4.3. Culture Media

For the enrichment cultures and biosurfactant screening of the strains, a slightly modified Bushnell–Haas medium (BH) [45] was used: (g/L); MgSO_4_—0.2; CaCl_2_—0.02; KH_2_PO_4_—1; K_2_HPO_4_—1; NH_4_NO_3_—1; FeCl_3_ × 6H_2_O—0.05; NaCl—100; the pH of the medium was adjusted to 7 ± 0.2 with 1N NaOH. BH agar plates were prepared with the above-mentioned medium with added 2% agar. For routine maintenance, the strains were cultured on HM medium [46]: (g/L); NaCl—100; MgSO_4_ × 7H_2_O—10; CaCl_2_ × 2H_2_O—0.36; KCl—2; NaHCO_3_—0.06; NaBr—0.23; FeCl_3_ × 6H_2_O—0.001; peptone—5; yeast extract—10; glucose—1; the pH of the medium was adjusted to 7.2 with 1N NaOH.

### 4.4. Enrichment and Strain Isolation

Briefly, 5 g of saline mud was mixed with 5 mL 10% NaCl and vortexed for 3 min. This suspension was inoculated in 50 mL modified Bushnell–Haas medium separately amended with three different carbon sources (n-hexadecane, olive oil, or glycerol) at a concentration of 2%. The enrichment cultures were cultivated for 7 days at 35 °C and 150 rpm in a BioSan ES 20/60 orbital shaker (Riga, Latvia). After this period, 5 mL inoculum from the enrichment cultures were used for a second round of enrichment under the same conditions. After the second enrichment, 10-fold dilutions in the range from 10^−1^ to 10^−9^ were made with 10% NaCl as diluent. After that, 100 μL from each dilution were spread plated on BH agar plates with 2% glycerol as carbon source and cultivated for 7 days at 35 °C in a thermostat Chirana thermostat (Stara Tura, Slovakia). The isolates were routinely cultured on HM agar plates.

### 4.5. Biosurfactant Screening

In glass test tubes, one colony from each strain was inoculated in 5 mL BH medium with 2% glycerol. The cultures were incubated for 7 days at 35 °C and 180 rpm. Then, cell-free supernatants (CFSs) were obtained by centrifugation at 13,400 rpm for 5 min. The CFSs were filter-sterilised through 0.2 μm syringe filter. The obtained CFSs were screened for possible biosurfactant production from the isolates by several methods.

The haemolytic activity of the CFSs was tested with 5% blood agar plates [4]. Briefly, 6 mm wells were made in the blood agar by a sterile cork borer and 100 μL CFS from each strain were pipetted. The plates were incubated at 37 °C for 24 h and then observed for haemolytic halos.

An oil displacement test was conducted by spreading 2 mL olive oil stained with Sudan black III on the surface of 20 mL dH_2_O poured in a Petri dish [47]. After that, 20 μL CFS were dropped onto the surface of the olive oil, and the diameter of the oil-displacement zone was measured in mm.

Parafilm M assay was also conducted by placing a 10 μL drop of CFS onto the surface of Parafilm M used as a hydrophobic surface [34]. Then, the shape and diameter of the drop were observed. In the presence of biosurfactant in the CFS, the drop would be flat with larger diameter while a dome shaped drop with narrower diameter indicates absence of biosurfactant. Based on the shape of the drop, the results were interpreted as positive or negative.

Emulsifying activity of the CFS was assessed by emulsifying index (E_24_) [48]. In glass test tubes, 1 mL CFS was mixed with 1 mL triolein and vortexed at maximal speed for 2 min. The resulting emulsion was incubated at 20 °C for 24 h. The E_24_ index was calculated as the height of the emulsion layer divided by the total height of the mixture, measured in mm, and multiplied by 100.

### 4.6. Surface Tension Measurement

The surface tension measurements of the obtained CFS were conducted using Force Tensiometer K20 (KRÜSS, Hamburg, Germany), based on the DuNouy ring method [49]. All experiments are performed at room temperature (T = 23 ± 1 °C) after about 30 min temperature equilibration of the measured sample.

### 4.7. 16S rRNA Gene Sequencing

After the biosurfactant screening, four isolates were chosen as the most prominent for biosurfactant production. From each of these strains total genomic DNA was extracted with a commercial GenEluteTM Bacterial Genomic DNA Kit (Merck KGaA, Darmstadt, Germany), applying the manufacturer’s instructions. After the DNA extraction, the 16S rRNA gene was amplified by PCR using universal bacterial primers 8F (5′-AGAGTTTGATCCTGG CTCAG-3′) and 1513R (5′-TACGTTACCTTGTTACGA CTT-3′) (SciTechS). BioRAD T100^®^ Thermal Cycler (Bio-Rad Laboratories, Inc., Hercules, CA, USA) was used for conducting the PCR and AccuPower^®^ PCR PreMix and Master Mix (Bioneer, Yuseong-gu, Daejeon 34013, Republic of Korea) was used following amplification protocol that was previously mentioned [50]. A 1% agarose gel electrophoresis was conducted with an electrophoretical system Consort E122 (Consort bvba, Turnhout, Belgium) to verify the presence and integrity of the extracted total DNA and amplified 16S rRNA gene PCR products. The obtained 16S rRNA gene PCR products were sequenced with 27F (5′AGAGTTTGATCMTGGCTCAG-3′) and 1492R (5′ACCTTGTTACGACTT-3′) primers by Macrogen Inc., Seoul, Republic of Korea.

### 4.8. Identification of the Strains and Phylogenetic Analysis

The strains were taxonomically identified based on their 16S rRNA gene sequence via a BLAST 2.17.0 [51] search using the NCBI platform [52]. The obtained sequences were compared to the deposited sequences in GenBank database in order to find their closest phylogenetic relatives with sequence similarity ≥98%. After that, all the sequences were retrieved and used for multiple sequence alignment with ClustalW algorithm. All aligned sequences were subjected to phylogenetic analysis in MEGA 11.0.13 [53]. Their evolutionary distance was assessed in units measuring the number of base substitutions per site with the Tamura-Nei method [54]. The neighbour-joining method [55] was used to reconstruct the tree and the statistical significance of the topology of the tree was computed based on 1000 bootstrap replications, while the bootstrap values were shown above the branches [56]. The rate variation among sites was modelled with a gamma distribution (shape parameter = 5). *Cobetia marina* DSM 4741^T^ (NR 042065.1) was used as an outgroup in order to root the tree. The 16S rRNA gene sequences of the four chosen strains were deposited in GenBank database under the following accession numbers: BS7OL—PX488313.1, BS8OL—PX488314.1, BS9GL—PX488316.1, and BS10HD—PX488317.1.

### 4.9. Optimisation of Biosurfactant Production by Halomonas ventosae BS10HD

For all the experiments one colony of the strain was suspended in 500 μL 10% NaCl and this suspension was used as inoculum for 5 mL HM medium which was incubated for 24 h at 35 °C and 150 rpm. After cultivation, this pre-culture was used to inoculate 50 mL HM medium in 100 mL flask which was again incubated at the same conditions until OD_600_ of 2 was reached. This liquid culture was used as inoculum for all the subsequent experiments which were again conducted with 50 mL HM medium in 100 mL flasks. The initial OD_600_ of the liquid cultures was 0.1. The basal conditions for biosurfactant production by *Halomonas ventosae* BS10HD were 120 h, 10% NaCl 5% inoculum, 2% n-hexadecane as carbon source, NH_4_NO_3_ as nitrogen source, pH 7, 150 rpm, and 35 °C. Optimisation of the biosurfactant production was conducted by varying the fermentation process conditions one factor at a time while maintaining the others unchanged. The fermentation time (24 h, 48 h, 72 h, 96 h, 120 h), NaCl concentration (1%, 5%, 10%, 15%), inoculum volume (1%, 2%, 5%), pH of the medium (pH 6, pH 7, pH 8), nitrogen source type (NH_4_NO_3_, peptone, yeast extract), and carbon source type (n-hexadecane, olive oil, sunflower oil, liquid paraffin, glycerol) were varied. After cultivation, OD_600_ of the liquid cultures and E_24_ index of the obtained CFS were measured as indicators of the most suitable parameters for strain growth combined with biosurfactant production.

### 4.10. Growth of H. ventosae BS10HD and Biosurfactant Production

Biosurfactant production and *H. ventosae* BS10HD growth were monitored over time. The pre-culture that served as inoculum was prepared as described above. The time course of biosurfactant production and cell growth were determined in 100 mL flasks with 50 mL modified BH medium under optimized conditions over a period of 96 h. At regular intervals of time (6 h, 12 h, 24 h, 48 h, 72 h, and 96 h), the samples were withdrawn from the liquid culture and the OD_600_ and E_24_ indices were measured.

### 4.11. Biosurfactant Extraction

Bacterial cells were removed by centrifugation (6000× *g*, at 4 °C for 15 min). Culture supernatant was acidified with 6 N HCl to pH of 2.0, and was stored overnight at 4 °C. The CFS was extracted twice with different organic solvents (ethyl acetate, methylene chloride, and chloroform). The extraction time was 4 h for good separation of the aqueous and organic layers. The most effective solvent for biosurfactant recovery was determined using thin-layer chromatography (TLC). Based on the intensity and saturation of the spots observed on the TLC plates, methylene chloride was identified as the solvent yielding the highest recovery of the target biosurfactant. Another criterion for solvent selection was quantitative evaluation of the biosurfactant obtained.

The methylene chloride was added to the culture supernatant, and after being vigorously shaken, the mixtures were allowed to stand until phase separation. The organic phases were collected and evaporated to obtain the crude product.

### 4.12. Thin-Layer Chromatography (TLC)

Thin-layer chromatography (TLC) was carried out on silica gel 60 F254 TLC plates (Merck Millipore, MA, USA). The mobile phase comprised analytical-grade chloroform, methanol, and water in the ratio of 65:25:2 by volume. The spots were visualized with 2% anthrone-sulphate reagent, 2% ninhydrin/ethanol, and phosphomolybdic acid, followed by heating at 110 °C for 2 min.

### 4.13. Fourier-Transform Infrared Spectroscopy Analysis (FTIR)

FTIR spectra of the sample were recorded on a Bruker Invenio R spectrometer (Billerica, MA, USA), equipped with a detector of deuterated triglycinesulphate. The spectra were collected in the frequency region of 4000 cm^−1^ to 400 cm^−1^ with 128 scans at a resolution of 2 cm^−1^ by direct deposition of the samples on an attenuated total reflectance element (ATR), a diamond crystal. The chemical nature of biosurfactants was revealed by the detection of their functional groups.

### 4.14. Statistical Analysis

All experiments and analyses were performed in triplicate, and the data were expressed as mean values ± standard deviation. Statistical analysis was performed using Excel, Microsoft Office 365.

## 5. Conclusions

A new halophilic strain, BS10HD, was isolated from solar salterns and selected as the most potent biosurfactant producer. Its phylogenetic affiliation was determined by 16S rRNA gene sequence analysis, and according to the nucleotide sequence, it was assigned to *Halomonas ventosae*. It showed strong emulsifying activity (E_24_ = 56%) and reduced surface tension to 27.25 mN/m after 7 days of batch fermentation at optimized conditions and utilizing hexadecane as a source of carbon. Preliminary TLC and FTIR analyses suggest that the biosurfactant produced by *H. ventosae* BS10HD is a lipopeptide compound and further investigations are ongoing. The biosurfactants derivatives, such as glycolipids, lipopeptides, glycoproteins, surfactins, rhamnolipids, and polymers, have encouragingly low toxicology profiles, with potential in important health and remediation applications.

## Figures and Tables

**Table 1:** Biosurfactant screening of the CFSs obtained from the isolates.

Strain	Oil Displacement (mm)	Haemolysis Halo Zone (mm)	Parafilm M Assay	E_24_, (%)
BS1GL	30	5	+	43.75
BS2GL	21	10	−	50
BS3GL	24	−	−	52.94
BS4GL	22	−	+	43.75
BS5GL	20	5	+	43.75
BS6GL	20	8	−	52.94
BS7GL	18	−	+	50
BS8GL	17	5	+	50
BS9GL	22	−	−	56.25
BS10GL	21	5	+	43.75
BS1OL	11	−	+	50
BS2OL	18	−	−	43.75
BS3OL	3	7	−	ND
BS4OL	2	6	−	ND
BS5OL	5	7	−	ND
BS6OL	15	5	+	43.75
BS7OL	11	−	−	56.25
BS8OL	21	−	−	56.25
BS9OL	18	5	−	50
BS10OL	12	−	−	ND
BS1HD	5	7	−	50
BS2HD	30	−	+	50
BS3HD	2	7	−	ND
BS4HD	2	7	−	ND
BS5HD	2	7	−	ND
BS6HD	2	7	−	ND
BS7HD	2	7	−	ND
BS8HD	3	8	−	ND
BS9HD	2	7	−	ND
BS10HD	10	6	−	56.25

**Table 2:** Taxonomic identification of the four prioritized strains based on the 16S rRNA gene sequence analysis and sequence similarity to their closest phylogenetic relative deposited in the GenBank database.

Strain	Closest Relative in Genbank	Accession Number	Sequence Length (bp)	Sequence Similarity (%)
BS7OL	*Halomonas koreensis* SS20^T^ (NR 025773.1)	PX488313.1	1380	98.97%
BS8OL	*Gracilibacillus massiliensis* Awa-1^T^ (NR 149182.1)	PX488314.1	1416	99.08%
BS9GL	*Thalassobacillus devorans* G-19.1^T^ (NR 042273.1)	PX488316.1	1382	99.20%
BS10HD	*Halomonas ventosae* Al12^T^ (NR 042812.1)	PX488317.1	1369	99.27%

## Data Availability

Available upon request.

## References

[1] Karanth N.G.K., Deo P.G., Veenanadig N.K. (1999). Microbial Production of Biosurfactants and Their Importance. Curr. Sci..

[2] Dias M.A.M., Nitschke M. (2023). Bacterial-Derived Surfactants: An Update on General Aspects and Forthcoming Applications. Braz. J. Microbiol..

[3] Eras-Muñoz E., Farré A., Sánchez A., Font X., Gea T. (2022). Microbial Biosurfactants: A Review of Recent Environmental Applications. Bioengineered.

[4] Tiso T., Germer A., Küpper B., Wichmann R., Blank L.M., McGenity T.J., Timmis K.N., Nogales Fernández B. (2015). Methods for Recombinant Rhamnolipid Production. Hydrocarbon and Lipid Microbiology Protocols.

[5] Simões C.R., Da Silva M.W.P., De Souza R.F.M., Hacha R.R., Merma A.G., Torem M.L., Silvas F.P.C. (2024). Biosurfactants: An Overview of Their Properties, Production, and Application in Mineral Flotation. Resources.

[6] Schultz J., Rosado A.S. (2020). Extreme Environments: A Source of Biosurfactants for Biotechnological Applications. Extremophiles.

[7] Kochhar N., I.K K., Shrivastava S., Ghosh A., Rawat V.S., Sodhi K.K., Kumar M. (2022). Perspectives on the Microorganism of Extreme Environments and Their Applications. Curr. Res. Microb. Sci..

[8] Marzban G., Tesei D. (2025). The Extremophiles: Adaptation Mechanisms and Biotechnological Applications. Biology.

[9] Salas-Bruggink D.I.J., Martín J.S.-S., Leiva G., Blamey J.M. (2024). Extremozymes: Challenges and Opportunities on the Road to Novel Enzymes Production. Process Biochem..

[10] Alvionita M., Hertadi R. (2019). Bioconversion of Glycerol to Biosurfactant by Halophilic Bacteria *Halomonas elongata* BK-AG18. Indones. J. Chem..

[11] Corti Monzón G., Nisenbaum M., Herrera Seitz M.K., Murialdo S.E. (2018). New Findings on Aromatic Compounds’ Degradation and Their Metabolic Pathways, the Biosurfactant Production and Motility of the Halophilic Bacterium *Halomonas* sp. KHS3. Curr. Microbiol..

[12] Dhasayan A., Kiran G.S., Selvin J. (2014). Production and Characterisation of Glycolipid Biosurfactant by *Halomonas* sp. MB-30 for Potential Application in Enhanced Oil Recovery. Appl. Biochem. Biotechnol..

[13] Pepi M., CesÃro A., Liut G., Baldi F. (2005). An Antarctic Psychrotrophic Bacterium *Halomonas* sp. ANT-3b, Growing on n-Hexadecane, Produces a New Emulsyfying Glycolipid. FEMS Microbiol. Ecol..

[14] Donio M.B.S., Ronica F.A., Viji V.T., Velmurugan S., Jenifer J.S.C.A., Michaelbabu M., Dhar P., Citarasu T. (2013). *Halomonas* sp. BS4, A Biosurfactant Producing Halophilic Bacterium Isolated from Solar Salt Works in India and Their Biomedical Importance. SpringerPlus.

[15] Mnif S., Chamkha M., Sayadi S. (2009). Isolation and Characterization of *Halomonas* sp. Strain C2SS100, a Hydrocarbon-Degrading Bacterium under Hypersaline Conditions. J. Appl. Microbiol..

[16] Donio M., Ronica S., Viji V.T., Velmurugan S., Jenifer J.A., Michaelbabu M., Citarasu T. (2013). Isolation and Characterization of Halophilic *Bacillus* sp. BS3 Able to Produce Pharmacologically Important Biosurfactants. Asian Pac. J. Trop. Med..

[17] Tripathi L., Twigg M.S., Zompra A., Salek K., Irorere V.U., Gutierrez T., Spyroulias G.A., Marchant R., Banat I.M. (2019). Biosynthesis of Rhamnolipid by a *Marinobacter* Species Expands the Paradigm of Biosurfactant Synthesis to a New Genus of the Marine Microflora. Microb. Cell Fact..

[18] Peng F., Liu Z., Wang L., Shao Z. (2007). An Oil-Degrading Bacterium: *Rhodococcus erythropolis* Strain 3C-9 and Its Biosurfactants. J. Appl. Microbiol..

[19] Fazli R.R., Hertadi R. (2019). Production and Characterization of Rhamnolipids from Bioconversion of Palm Oil Mill Effluent by the Halophilic Bacterium *Pseudomonas stutzeri* BK-AB12. Env. Prog. and Sustain. Energy.

[20] Gaur S., Gupta S., Jain A. (2021). Characterization and Oil Recovery Application of Biosurfactant Produced During Bioremediation of Waste Engine Oil by Strain *Pseudomonas aeruginosa* gi|KP 16392| Isolated from Sambhar Salt Lake. Biorem. J..

[21] Haque E., Riyaz M.A.B., Shankar S., Hassan S. (2020). Physiochemical and Structural Characterization of Biosurfactant Produced by Halophilic *Pseudomonas aeruginosa* ENO14 Isolated from Seawater. Int. J. Pharm. Investig..

[22] Citarasu T., Thirumalaikumar E., Abinaya P., Babu M.M., Uma G. (2021). Biosurfactants from Halophilic Origin and Their Potential Applications. Green Sustainable Process for Chemical and Environmental Engineering and Science.

[23] Pradhan A.K., Pradhan N., Mall G., Panda H.T., Sukla L.B., Panda P.K., Mishra B.K. (2013). Application of Lipopeptide Biosurfactant Isolated from a Halophile: *Bacillus tequilensis* CH for Inhibition of Biofilm. Appl. Biochem. Biotechnol..

[24] Essghaier B., Naccache C., Ben-Miled H., Mottola F., Ben-Mahrez K., Mezghani Khemakhem M., Rocco L. (2024). Discovery and Characterization of Novel Lipopeptides Produced by *Virgibacillus massiliensis* with Biosurfactant and Antimicrobial Activities. 3 Biotech.

[25] Truskewycz A., Gundry T.D., Khudur L.S., Kolobaric A., Taha M., Aburto-Medina A., Ball A.S., Shahsavari E. (2019). Petroleum Hydrocarbon Contamination in Terrestrial Ecosystems—Fate and Microbial Responses. Molecules.

[26] Kim M., Oh H.-S., Park S.-C., Chun J. (2014). Towards a Taxonomic Coherence Between Average Nucleotide Identity and 16S rRNA Gene Sequence Similarity for Species Demarcation of Prokaryotes. Int. J. Syst. Evol. Microbiol..

[27] Barale S.S., Ghane S.G., Sonawane K.D. (2022). Purification and Characterization of Antibacterial Surfactin Isoforms Produced by Bacillus Velezensis SK. AMB Expr..

[28] Meena K.R., Sharma A., Kumar R., Kanwar S.S. (2020). Two Factor at a Time Approach by Response Surface Methodology to Aggrandize the Bacillus Subtilis KLP2015 Surfactin Lipopeptide to Use as Antifungal Agent. J. King Saud Univ. Sci..

[29] Zhao X., Zhou Z., Han Y. (2017). Antifungal Effects of Lipopeptide Produced by *Bacillus amyloliquefaciens* BH072. Adv. Biosci. Biotechnol..

[30] Imhoff J.F., Labes A., Wiese J. (2011). Bio-Mining the Microbial Treasures of the Ocean: New Natural Products. Biotechnol. Adv..

[31] Pacwa-Płociniczak M., Płaza G.A., Piotrowska-Seget Z., Cameotra S.S. (2011). Environmental Applications of Biosurfactants: Recent Advances. Int. J. Mol. Sci..

[32] Fariq A., Yasmin A. (2020). Production, Characterization and Bioactivities of Biosurfactants from Newly Isolated Strictly Halophilic Bacteria. Process Biochem..

[33] Vater J., Kablitz B., Wilde C., Franke P., Mehta N., Cameotra S.S. (2002). Matrix-Assisted Laser Desorption Ionization-Time of Flight Mass Spectrometry of Lipopeptide Biosurfactants in Whole Cells and Culture Filtrates of *Bacillus subtilis* C-1 Isolated from Petroleum Sludge. Appl. Environ. Microbiol..

[34] Kuiper I., Lagendijk E.L., Pickford R., Derrick J.P., Lamers G.E.M., Thomas-Oates J.E., Lugtenberg B.J.J., Bloemberg G.V. (2004). Characterization of Two *Pseudomonas putida* Lipopeptide Biosurfactants, Putisolvin I and II, Which Inhibit Biofilm Formation and Break down Existing Biofilms. Mol. Microbiol..

[35] Cheffi M., Maalej A., Mahmoudi A., Hentati D., Marques A.M., Sayadi S., Chamkha M. (2021). Lipopeptides Production by a Newly *Halomonas venusta* Strain: Characterization and Biotechnological Properties. Bioorg. Chem..

[36] Nurfarahin A.H., Mohamed M.S., Phang L.Y. (2018). Culture Medium Development for Microbial-Derived Surfactants Production—An Overview. Molecules.

[37] Almuhayawi M.S., Elshafey N., Hagagy N., Selim S., Al Jaouni S.K., Sofy A.R., Samy M., Gattan H.S., Alruhaili M.H., Alharbi M.T. (2024). Exploring Biosurfactant from *Halobacterium jilantaiense* as Drug Against HIV and Zika Virus: Fabrication, Characterization, Cytosafety Property, Molecular Docking, and Molecular Dynamics Simulation. Front. Bioeng. Biotechnol..

[38] Karbalaei-Heidari H., Taghavi L., Hasani Zadeh P. (2020). Induction of Biosurfactant Production from a Native Isolated Moderately Halophilic Bacterium, *Halomonas* sp. MM93 in the Presence of Olive Oil and Study of Its Stability. Modares J. Biotechnol..

[39] Cheffi M., Hentati D., Chebbi A., Mhiri N., Sayadi S., Marqués A.M., Chamkha M. (2020). Isolation and Characterization of a Newly Naphthalene-Degrading *Halomonas pacifica*, Strain Cnaph3: Biodegradation and Biosurfactant Production Studies. 3 Biotech.

[40] Janek T., Gudiña E.J., Połomska X., Biniarz P., Jama D., Rodrigues L.R., Rymowicz W., Lazar Z. (2021). Sustainable Surfactin Production by *Bacillus subtilis* Using Crude Glycerol from Different Wastes. Molecules.

[41] Ali N., Pang Z., Wang F., Xu B., El-Seedi H.R. (2022). Lipopeptide Biosurfactants from *Bacillus* spp.: Types, Production, Biological Activities, and Applications in Food. J. Food Qual..

[42] Carolin C.F., Kumar P.S., Ngueagni P.T. (2021). A Review on New Aspects of Lipopeptide Biosurfactant: Types, Production, Properties and Its Application in the Bioremediation Process. J. Hazard. Mater..

[43] Singh A., Van Hamme J.D., Ward O.P. (2007). Surfactants in Microbiology and Biotechnology: Part 2. Application Aspects. Biotechnol. Adv..

[44] Janek T., Łukaszewicz M., Rezanka T., Krasowska A. (2010). Isolation and Characterization of Two New Lipopeptide Biosurfactants Produced by *Pseudomonas fluorescens* BD5 Isolated from Water from the Arctic Archipelago of Svalbard. Bioresour. Technol..

[45] Bushnell L.D., Haas H.F. (1940). The Utilization of Certain Hydrocarbons by Microorganisms. J. Bacteriol..

[46] Ventosa A., Quesada E., Rodriguez-Valera F., Ruiz-Berraquero F., Ramos-Cormenzana A. (1982). Numerical Taxonomy of Moderately Halophilic Gram-Negative Rods. Microbiology.

[47] Morikawa M., Daido H., Takao T., Murata S., Shimonishi Y., Imanaka T. (1993). A New Lipopeptide Biosurfactant Produced by *Arthrobacter* sp. Strain MIS38. J. Bacteriol..

[48] Cooper D.G., Goldenberg B.G. (1987). Surface-Active Agents from Two *Bacilllus* Species. Appl. Environ. Microbiol..

[49] Su G. (2024). Review on Factors Affecting Nanofluids Surface Tension and Mechanism Analysis. J. Mol. Liq..

[50] Tomova I., Lazarkevich I., Tomova A., Kambourova M., Vasileva-Tonkova E. (2013). Diversity and Biosynthetic Potential of Culturable Aerobic Heterotrophic Bacteria Isolated from Magura Cave, Bulgaria. Int. J. Speleol..

[51] Altschul S.F., Gish W., Miller W., Myers E.W., Lipman D.J. (1990). Basic Local Alignment Search Tool. J. Mol. Biol..

[52] Sayers E.W., Bolton E.E., Brister J.R., Canese K., Chan J., Comeau D.C., Connor R., Funk K., Kelly C., Kim S. (2022). Database Resources of the National Center for Biotechnology Information. Nucleic Acids Res..

[53] Tamura K., Stecher G., Kumar S. (2021). MEGA11: Molecular Evolutionary Genetics Analysis Version 11. Mol. Biol. Evol..

[54] Tamura K., Nei M. (1993). Estimation of the Number of Nucleotide Substitutions in the Control Region of Mitochondrial DNA in Humans and Chimpanzees. Mol. Biol. Evol..

[55] Saitou N., Nei M. (1987). The Neighbor-Joining Method: A New Method for Reconstructing Phylogenetic Trees. Mol. Biol. Evol..

[56] Felsenstein J. (1985). Confidence Limits on Phylogenies: An Approach Using the Bootstrap. Evolution.

